# Identification and Validation of SAA4 as a Rheumatoid Arthritis Prescreening Marker by Liquid Chromatography Tandem-mass Spectrometry

**DOI:** 10.3390/molecules22050805

**Published:** 2017-05-14

**Authors:** AeEun Seok, Hyun-Jung Lee, Sungeun Lee, Jiyeong Lee, Sora Mun, Arum Park, Yeon-Tae Chun, Jae-Hyeon Lee, Hee-Joung Lim, Hee-Gyoo Kang

**Affiliations:** 1 Laboratory of Signal Transduction and Disease Biomarker Discovery, Department of Senior Healthcare, BK21 Plus Program, Graduate School, Eulji University, Daejeon 34824, Korea; anne3082@gmail.com (A.S.); sora6456@naver.com (S.M.); sksskdi5959@naver.com (A.P.); meetjeonyt@naver.com (Y.-T.C.); 2 Research Institute of DongDeok Pharmaceutical, Chungcheongbuk-do 27864, Korea; sungelee982@gmail.com (S.L.); hyeon9139@gmail.com (J.-H.L.); 3 Department of Biomedical Laboratory Science, College of Health Sciences, Eulji University, Seongnam-si, Gyeonggi-do 13135, Korea; poopilove@hanmail.net (H.-J.L.); bjssw@naver.com (J.L.); 4 Department of Laboratory Medicine, Korea Cancer Center Hospital, Seoul 01812, Korea; 5 Integrative Research Support Center, College of Medicine, The Catholic University of Korea, Seoul 06591, Korea; 6 Forensic Science R&D Lab., Police Science Institute, Chungcheongnam-do 31539, Korea

**Keywords:** rheumatoid factor, LC-MS/MS, pre-screening, serum amyloid A4

## Abstract

Rheumatoid arthritis (RA) is a chronic autoimmune disease that progresses into systemic inflammation and joint deformity. RA diagnosis is a complicated procedure, and early diagnostic methods are insufficient. Therefore, in this study, we attempted to identify new markers to improve the accuracy of RA prescreening. e identified differentially expressed proteins (DEPs) by using liquid chromatography tandem-mass spectrometry in health-prescreening sera with high rheumatoid factor (RF) values, and compared the findings with those from sera with normal RF values. We identified 93 DEPs; of these, 36 were upregulated, and 57 were downregulated in high-RF sera. Pathway analysis revealed that these DEPs were related to immune responses. Additionally, four DEPs were statistically analyzed by proteomic analysis; of these, SAA4 was significantly validated in individual enzyme-linked immunosorbent assays. Moreover, SAA4 was significantly upregulated in RA patients (n = 40, 66.43 ± 12.97 ng/mL) compared with normal controls (n = 40, 4.79 ± 0.95 ng/mL) and had a higher area under the curve than C-reactive protein. Thus, we identified SAA4 as a protein that was positively correlated with RF and RA. SAA4 may represent a novel prescreening marker for the diagnosis of RA.

## 1. Introduction

Early diagnosis and control of disease progression are important for the prevention of chronic diseases, particularly those that leave irreversible lesions, have high mortality rates, and reduce quality of life [1,2]. Accordingly, current diagnostic research is focused on improving patient quality of life through early diagnosis of chronic diseases using simple diagnostic methods. As a representative example of prescreening in chronic disease diagnosis, diabetes, which is characterized by high glucose concentrations and accompanied by various complications, can be rapidly and accurately diagnosed through blood and urine tests [3]. Emerging technologies in genomics and proteomics may have applications in increasing the specificity of prescreening and early detection in order to prevent disease occurrence.

Rheumatoid arthritis (RA) is a chronic autoimmune disease characterized by increased synovial membrane permeability and partial inflammation; RA eventually progresses to systemic inflammation and osteoarthritis accompanied with the generation of painful, irreversible lesions [4,5,6]. Finally, RA progresses to physical disability or mortality when appropriate treatment is not provided. The prevalence of RA is 0.5–1.3% of the adult population [7], and women are 2–3 times more likely to develop RA than men [8]. Indeed, Schellekens et al. reported that 70% of patients exhibit joint damage within 2 years after the onset of symptoms [2,9], and patients with RA have mortality rates that are twice as high as those of the general population [10,11,12]. However, current RA screening methods are complicated, and the initial diagnosis is difficult owing to the time- and cost-burden of diagnostic techniques.

The most common clinical test of RA is the detection of rheumatoid factor (RF), carried out by measuring autoantibodies against the Fc fragment of immunoglobulin G [13]. However, diagnosis based on RF alone is unreliable because RF levels are also increased in cases of chronic infection or immune-related diseases such as systemic lupus erythematosus [14]. Therefore, physicians generally diagnose RA by integrating the results of blood tests, duration of symptoms, and examination of swollen or tender joints, following the Rheumatoid Arthritis Classification Criteria (2010) [15]. In blood tests, measurements of C-reactive protein (CRP) and erythrocyte sedimentation rate (ESR) are conducted to detect inflammation; however, these markers are not specific for RA [16,17,18]. Anti-citrullinated protein antibodies (ACPAs) detect anti-citrullin and have better diagnostic rates, but are only present in 70–90% of patients with RA [9,19]. Alternative diagnostic approaches include genetic profiling of specific RA-related genes. Genetic profiling of autoimmune factors has also revealed the potential relevance of the HLA class II genotype, which may be related to RA progression; indeed, over 80% of patients with RA carry the HLA-DRB1*04 epitope [20,21].

Biomarkers are biological molecules/genes/characteristics that indicate the presence of a disease. Biomarkers include genes, proteins, blood or tissue metabolites, blood pressure, and pulse [22]. In particular, proteomics may provide individualized information directly related with differential protein change. A number of prescreening, diagnostic, and monitoring methods have been evaluated through protein analysis [23,24]. Following the development of tandem mass spectrometry (MS/MS) systems and related software, the specificity of protein analysis has improved dramatically, allowing researchers to obtain accurate information for clinical diagnosis, including information on protein abundance [25]. For example, CRP has been quantified in the blood of patients with RA through multiple-reaction monitoring (MRM) [26]. Thus, analysis of CRP with S100A8, S100A9, and S100A12 by liquid chromatography (LC)-MS/MS has been suggested as a diagnostic method [27]. In addition, leucine-rich α-2 glycoprotein (LRG) may be a potential marker in patients with RA [28]. However, all of these biomarkers do not specifically distinguish RA from other immune or inflammatory diseases and therefore, they have limited use as diagnostic markers of RA.

In this study, we applied proteomics, including LC-MS/MS and pathway analysis, to identify new marker candidates that could improve prescreening efficiency, combined with RF, in early health screening. Our results revealed that serum amyloid A4 (SAA4) might represent a novel prescreening marker for the diagnosis of RA.

## 2. Results

### 2.1. Identification of Proteins in Normal and RA Sera

**Figure 1:**
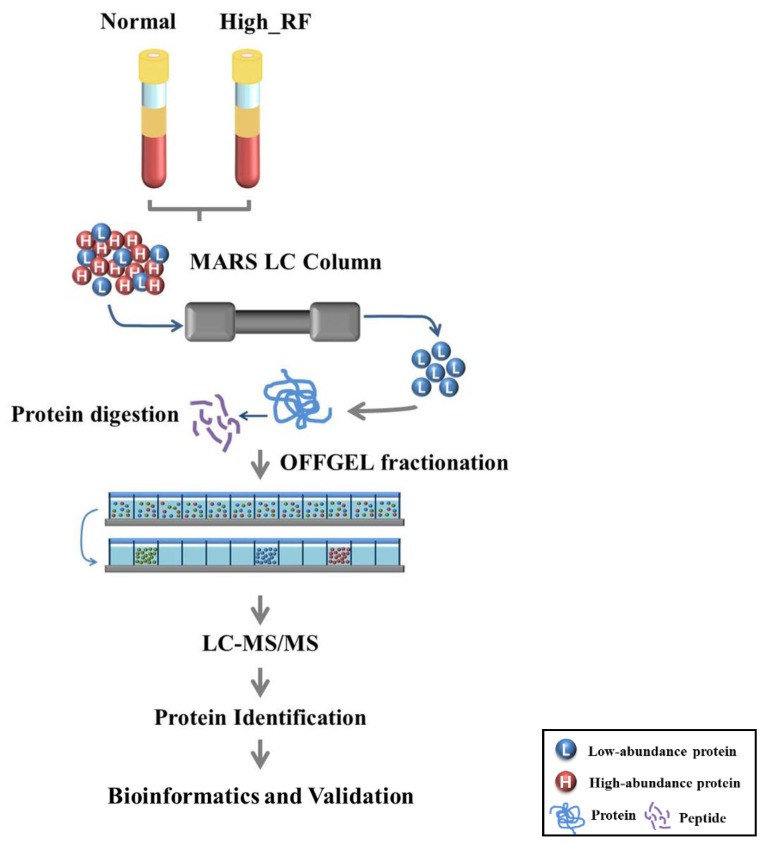
Experimental design adopted to identify novel diagnostic markers for rheumatoid arthritis (RA), using a proteomics-based approach.

**Figure 2:**
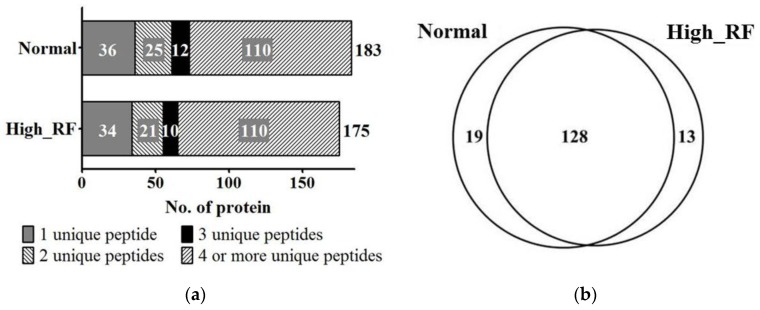

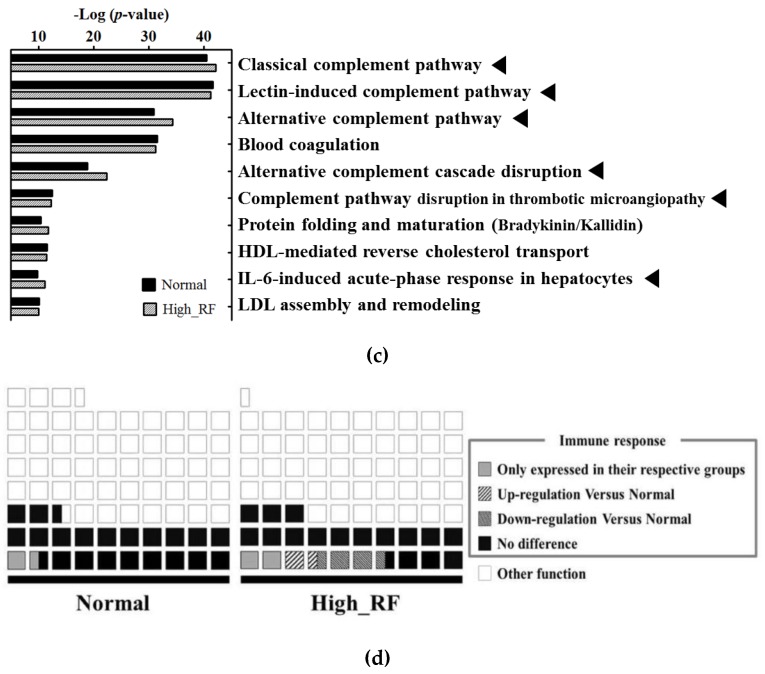
Proteomic profile of serum protein (normal rheumatoid factor [RF] versus high RF). (**a**) Number of identified proteins (147 [normal] and 141 [high RF] proteins were identified with two or more peptides). (**b**) Venn diagram depicting the protein overlap from (**a**) two or more peptides. (**c**) Pathway map of the identified proteins in RF-normal and -high sera. “◄” indicates processes involved in immune response. (**d**) Immune response-related proteins are depicted by various colors, while other functional proteins are marked in white. One white or colored square represents two identified proteins.

The experimental design is shown in Figure 1. From the nano-LC-MS/MS analysis, 1641 and 1709 peptide sequences were detected in the normal (RF value < 18 IU/mL) and high-RF groups (RF value > 18 IU/mL), respectively, by sequencing analysis (Supplementary Tables S1 and S2). To improve the reliability of the results, we discarded 36 and 34 identified proteins from 183 normal and RA samples, respectively, including one unique peptide from the normal and high-RF groups, from the protein list (Figure 2a). Protein analysis with two or more unique peptides identified 147 proteins in normal serum and 141 proteins in high RF-value serum. Moreover, 128 proteins were commonly expressed in both groups (Figure 2b).

### 2.2. Protein Network and Pathway Analysis

We analyzed the identified proteins using the GeneGo Metacore analysis program, to create protein pathway maps for each group. The 10 most common pathway maps were sorted according to statistical significance. Among the 10 networks, six were relevant to the immune response (Figure 2c), including 45 and 46 identified proteins in each group. Specifically, three of 46 immune-response proteins were upregulated, whereas six of 46 immune-response proteins were downregulated in the high RF-value sera compared with the normal sera. In addition, three and four proteins were unique to the normal and high RF-value groups, respectively (Figure 2d).

### 2.3. Comparative Analysis of the Expression Levels of the Identified Proteins

**Figure 3:**
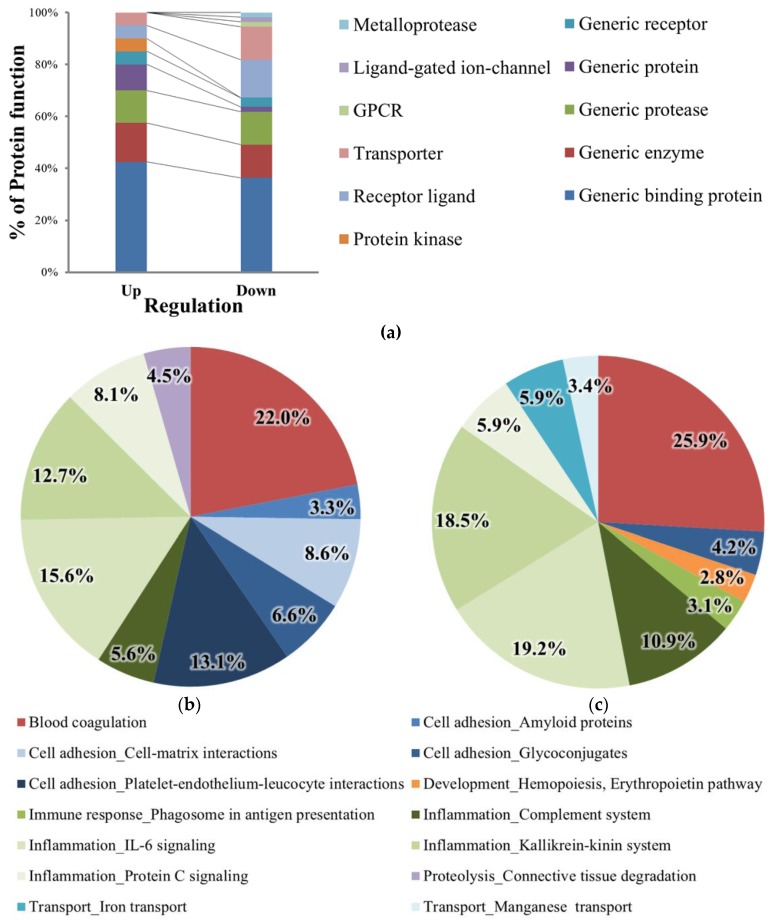
Functional analysis of proteins exhibiting at least a two-fold change in expression level in the high-RF group versus the normal group. (**a**) Functions of proteins exhibiting at least a two-fold change. (**b** and **c**) Process network of differentially expressed proteins (DEPs; up- or downregulated) in the high-RF group.

The data were then analyzed using Mass Profiler Professional (MPP) to confirm protein expression changes. The cut-off value for the comparative analysis of protein expression was a two-fold change in MPP analysis. Comparative analysis of the identified proteins revealed that 93 proteins were differentially expressed (Tables S3 and S4). Of these, 57 were downregulated and were functionally categorized as receptor ligands, transporters, G protein-coupled receptors (GPCRs), ligand-gated ion channels, and metalloproteases (Figure 3a). Additionally, 36 of these proteins were upregulated in the high RF-value sera and were related to cell adhesion and proteolysis.

### 2.4. Protein Network Process Analysis and Marker Candidate Selection

**Figure 4:**
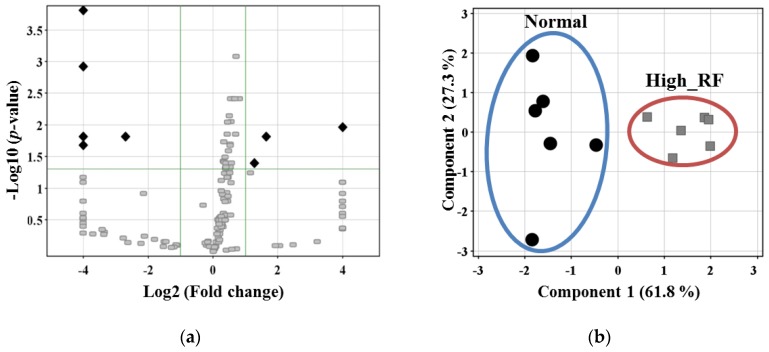
Mass Profiler Professional (MPP) analysis in the normal versus high-RF groups. (**a**) Volcano plot of DEPs. Thresholds are shown as gray lines. ♦: Proteins are significantly different in the volcano plot. (**b**) Principal component analysis (PCA) plot was calculated using the pooled sera of selected proteins from (**a**). The six black circles represent normal pooled sera, and the six grey squares represent high-RF pooled sera. Normal and high-RF clustered separately.

Next, we determined the top 10 process networks sorted according to statistical significance. Marked changes were observed for immune response or inflammation in the process network of DEPs in the high-RF group. The process network of upregulated proteins was associated with cell adhesion (Figure 3b), whereas that of downregulated proteins was involved in transport (Figure 3c). We selected eight proteins that showed greater than two-fold changes in expression between the normal and high-RF groups (*p* < 0.05; Figure 4a). Table 1 lists the three upregulated and five downregulated proteins. Then, from among the eight proteins, we excluded immunoglobulins, which should have been removed by the MARS column. Finally, four proteins remained, and principal component analysis (PCA) plots were generated to visualize the differences between the groups (Figure 4b).

### 2.5. Validation of SAA4 by Individual Serum Enzyme-linked Immunosorbent Assay (ELISA)

**Figure 5:**
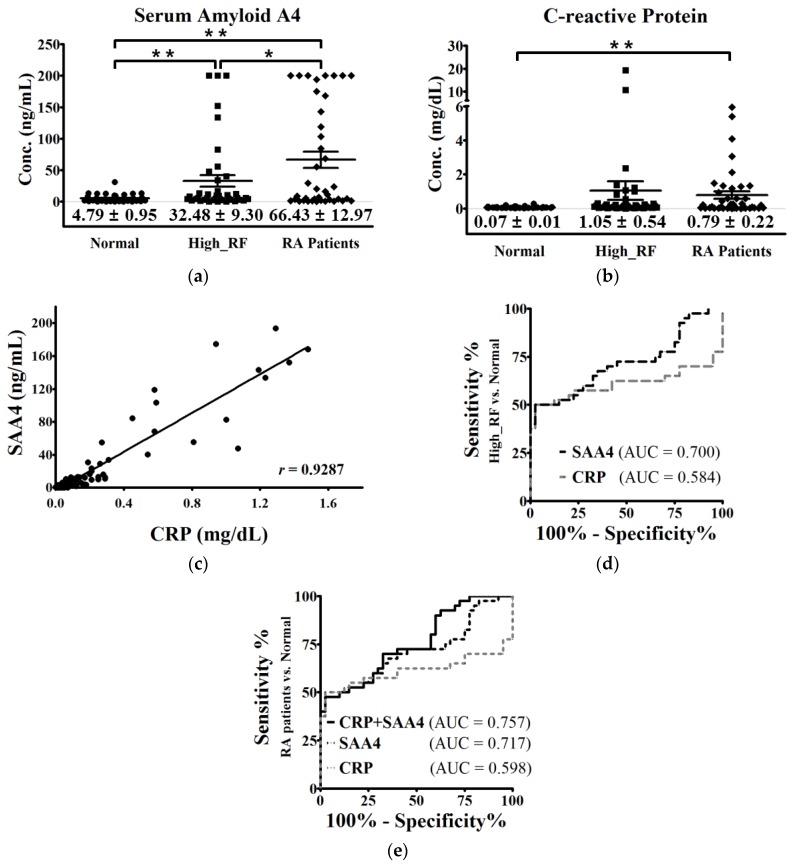
Validation of the biomarker candidate proteins for RA in the normal, high-RF, and RA diagnosed patient groups. Enzyme-linked immunosorbent assay (ELISA)-based quantification of (**a**) serum amyloid A4 (SAA4) and (**b**) c-reactive protein (CRP). Error bars indicate means ± standard errors (SEs). (**c**) The SAA4 levels correlated well with CRP levels in the sera from patients with RA (Pearson′s *r* = 0.9287, *p*-value < 0.001). (**d**) Receiver operating characteristic (ROC) curves and corresponding area under curve (AUC) values for SAA4 and CRP (0.700 and 0.584, respectively) in high-RF versus normal controls. (**e**) ROC and AUC values for SAA4, CRP, and combination of SAA4 with CRP (0.717, 0.598, and 0.757, respectively) in RA patients versus normal controls. * *p* < 0.05, ** *p* < 0.01.

We performed ELISAs to calculate the amount of differentially expressed proteins in each group. We chose three proteins for validation: cholinesterase (BCHE), leucine-rich alpha-2-glycoprotein (LRG1), and SAA4. However, our analysis of BCHE and LRG1 revealed that these proteins were not differentially expressed (data not shown). SAA4 was significantly upregulated in serum samples in the high RF-value and RA groups compared with the normal group (Figure 5a). CRP was also upregulated in the RA group, but the difference compared with the normal group was not significant (Figure 5b). Notably, there was a positive correlation between CRP and SAA4 in the RA group (r = 0.9287; Figure 5c). The receiver operating characteristic (ROC) curves of CRP and SAA4 in the high-RF and normal groups were analyzed and were found to have AUC values of 0.584 and 0.700 respectively (Figure 5d). And the high relevance of SAA4 and SAA4 with CRP were validated in the RA diagnosed patients and normal groups. The AUC values of CRP and SAA4 were 0.598 and 0.71(Figure 5e). Thus, SAA4 appeared to be superior to CRP as an RA marker based on the results of the ROC analysis. Moreover, the combined AUC value for SAA4 and CRP was 0.757, which was higher than those obtained in the independent analysis (Figure 5e).

## 3. Discussion

This study was designed to identify new candidate markers to increase the specificity of prescreening RA diagnosis using RF values. Accordingly, we used the RF value as a compartmentalized standard in the sample group for LC-MS/MS analysis, and four proteins, including SAA4, were identified as potential candidates.

SAA4 is a member of the constitutive serum amyloid A (SAA) isotype. Four subtypes of SAA have been identified to date; these subtypes are closely associated with in vivo cholesterol control in tissues and serum [29,30,31]. SAA1 and SAA2 are increased in the acute phase of inflammation by up to 1000-fold and are related to proteins involved in angiogenesis factor regulation, such as intracellular adhesion molecule 1 (ICAM-1), vascular cell adhesion molecule 1 (VCAM-1), and matrix metalloproteinase 1 (MMP1), in RA [32]. SAA has been reported to be valuable as a disease activity marker in the treatment of RA [33]. In addition, SAA is involved in joint destruction via MMP [34], and induced angiogenesis [35]. SAA3 is a member of the SAA family as well, but is considered a pseudo-gene. However, SAA3 was recently shown to have a role in the recovery of MMPs, as well as in promoting joint inflammation or destruction [36]. In contrast, the role of SAA4, which was shown to be differentially expressed in this study, is not well understood in human disease. SAA4 is expressed in graft-versus-host disease (GVHD) and epithelial ovarian tumors [37,38]; however, to the best of our knowledge, this is the first report describing SAA4 expression in RA. Moreover, we found that SAA4 was differentially expressed in RA, as detected by LC-MS/MS analysis of prescreening serum and validated by ELISA. Thus, SAA4 is clearly related to RF and may have applications as a new marker in prescreening approaches.

The SAA family of proteins are typical inflammatory factors in diseases [31]; therefore, it is difficult to determine what diseases or phenomena may trigger changes in SAA4 expression. In addition, data describing the role of SAA4 in other diseases are limited, and whether SAA4 has applications as an individual marker in RA diagnosis has not been investigated. In the current study, we confirmed the differential expression of SAA4 in patients with RA (Figure 5A). Currently, CRP is broadly used as an inflammation marker in RA pre-diagnosis [18,26]. However, it is also used as a diagnostic marker in various inflammation-related conditions, including vascular disease and cancer [16,39,40]. Our analysis suggested that SAA4 was superior to CRP in singular analysis in patients with RA (Figure 5D). Notably, the AUC of SAA4 combined with CRP was more efficient than the individual singlet tests for SAA4 and CRP. Use of SAA4 in combination with CRP may improve the pre-screening efficiency and accuracy of RA diagnosis (from 59.8% to 75.7%, Figure 5d). These data indicate that SAA4 may be a novel prescreening marker for early RA detection.

Proteomics-based approaches have applications in many fields and provide large amounts of data to medical sciences. In recent decades, proteomic approaches have been applied to identify new diagnostic markers or treatment indicators. Protein expression in tissues can reflect the health and disease status of the individual and provide information on prior conditions or predict future states. In this study, we found four specific RF-related proteins by proteomic analysis. However, all but one (SAA4) did not show significance in ELISA validation (data not shown). The failure of the ELISA-based validation may be explained by differences in the experimental methods and analytical parameters between LC-MS/MS and ELISA. Additionally, we used prescreening samples selected based on RF values to identify RF-related prescreening markers and pooled samples from the same group. This method may be applicable for final diagnosis and could limit differences. However, this blind analysis may also identify other diseases, different nutritional states, or medical histories; therefore, the validation may fail when analyzing individual samples. Interestingly, two of these failed targets, i.e., BCHE and LRG, have been previously reported to be associated with RA, albeit with conflicting results [28,41,42]. In a proteomics study of LRG1, increased expression of LRG1 was reported using isobaric tags for relative and absolute quantitation (iTRAQ)-based analysis. Moreover, other autoimmune diseases, such as Behcet′s disease (BD) and Crohn′s disease (CD), are also associated with increased LRG1 expression [28]. Another explanation for the failed validation of BCHE and LRG may be that these proteins are differentially expressed during different stages of RA progression. RA progression varies in each patient, and stage-specific variations in protein levels have been observed in RA [42].

## 4. Materials and Methods

### 4.1. Ethics Statement and Serum Collection

This study was approved by the Institutional Review Board of Korea Cancer Center Hospital (K-1408-002-069) and Eulji Hospital (EMC 2016-03-019). Serum samples were collected from the Korea Institute of Radiological and Medical Sciences (KIRAMS) Radiation Biobank (KRB) and Eulji Hospital. Thirty-six high-RF sera (RF value > 18 IU/mL, abnormal) were selected from among 90 randomly collected serum samples. We then sorted 18 normal sera samples according to the RF level (RF value < 18 IU/mL), and compared the clinical and laboratory data between the two groups (Table 2). Samples were pooled to sub-groups, and each group included sera from nine individuals, for MS/MS analysis. Each sub-group pooled sera sample was analyzed three-times by LC-MS/MS. Sera were individually validated using ELISA. Normal (n = 40) and high RF-value (n = 40) sera selected according to RF value, as well as sera from RA patients (n = 40), were used for validation.

### 4.2. Serum Depletion Using a Multiple Affinity Removal System LC Column

Before the MS/MS analysis, six high-abundance proteins (HAPs; i.e., albumin, IgG, IgA, transferrin, haptoglobin, and antitrypsin) were removed from six human serum samples to collect low-abundance proteins (LAPs). A multiple affinity removal system column (human 6-HC, 4.6 × 50 mm; Agilent Technologies, Santa Clara, CA, USA) was used for HAP removal, and the flow-through fractions were concentrated using a Pierce concentrator (7 mL/9 K; Thermo Scientific, Rockford, IL, USA) and a vacuum dryer (Scan Vac, LaboGene, Lynge, Denmark). Powdered LAPs were dissolved in 8 M urea for MS/MS analysis.

### 4.3. MS/MS Sample Preparation and Tryptic Digestion

The final concentration of LAPs dissolved in urea was measured by using Bradford assays (Bio-Rad, Hercules, CA, USA). One milligram of each LAP was then added to a new tube for reduction and alkylation. LAPs were incubated with 5 mM Tris (2-carboxyethyl) phosphine (Pierce, Rockford, IL, USA) and 15 mM iodoacetamide (Sigma-Aldrich, St. Louis, MO, USA) as described previously [40]. After reduction and alkylation, proteins were digested by using Trypsin Gold, mass spectrometry grade (Promega, Madison, WI, USA) at 37 °C, overnight. A C18 cartridge (Waters, Milford, MA, USA) was used to clean the final peptide. Final peptide solutions were dried in a vacuum dryer and then dissolved with double-distilled (dd) H_2_O for OFFGEL fractionation.

### 4.4. Peptide Fractionation by OFFGEL Electrophoresis

To maximize peptide detection, LAPs were separated to five isoelectric points using an OFFGEL Fractionator (3100 OFFGEL Low Res Kit, pH 3–10; Agilent Technologies). Peptide fractions from overnight OFFGEL separation were cleaned up using micro spin columns (Harvard Apparatus, Holliston, MA, USA) and then vacuumed dry for the next step.

### 4.5. Nano-LC-MS/MS

LAP fractions were analyzed using a high-performance liquid chromatography (HPLC)-chip/quadrupole time-of-flight (Q-TOF) system (Agilent Technologies). The specifications for the HPLC-Chip were as follows: 360-nL enrichment column; 75 μm × 150 mm separation column packed with Polaris C18-A (3 μm); sample running time, 120 min; flow rate, 0.3 μL/min.

The Q-TOF mass spectrometer was used in the positive ionization mode, and the drying nitrogen gas flow was set to 3 L/min with a temperature of 300 °C. Column-eluted peptides were selected for dissociation in the MS or MS/MS scan over the *m*/*z* range of 300–2400 or 100–3000, with a scan rate of 3.99 spectra/s in a collision cell. The isolation window was 4 *m*/*z*.

### 4.6. Protein Identification

The information regarding peptide sequences obtained in the previous step was analyzed using Spectrum Mill MS Proteomic Workbench Rev B.04.00.127 (Agilent Technologies) with the UniProKB/SWISSProt database (released in July 2016, Homo sapiens). Triplicate MS/MS runs were analyzed with the following parameters: precursor mass tolerance, 20 ppm; product ion mass tolerance, 50 ppm; maximum ambiguous precursor charge, 3; two missed cleavages allowed; digested by trypsin; fixed modification of carbamidomethyl cysteine and N-terminal carbamylation; variable modifications of oxidized methionine. After MS/MS searching, autovalidation was carried out by calculating the false-discovery rate (FDR). The threshold of the FDR was 1.2.

### 4.7. Label-free Quantification and Bioinformatics Analysis

The data were then exported to .txt file format for statistical analysis and visualized using the Mass Profiler Professional software (MPP; Agilent Technologies). Unpaired *t*-test with Benjamini-Hochberg false discovery rate (FDR) was executed to calculate the *p*-value. GeneGo Metacore (ISB, Seattle, WA, USA) was used to analyze protein functions, and a pathway map was generated.

### 4.8. ELISA

ELISA was conducted for the validation of DEPs. A commercially available sandwich ELISA kit (Cusabio, Wuhan, China) was used to measure the identified serum proteins, excluding the immunoglobulin family proteins. The experiment was performed as per the manufacturer′s instructions.

### 4.9. Statistical Analysis

ROC curve analysis was conducted to evaluate the usefulness of candidates with regard to high RF values. Areas under the ROC curve (AUCs) that were close to 1 had high power to distinguish the two populations (normal RF value versus RA). Two-tailed *t*-tests were used to analyze ROC curves, correlations, and ELISA validations. The statistical analyses were conducted using PRISM 5 (GraphPad Software, San Diego, CA, USA) and Statistical Package for Social Sciences version 16.0 (SPSS; SPSS Inc., Chicago, IL, USA).

## 5. Conclusions

In summary, in this study, we compared serum samples with normal and high RF values by proteomic analysis. We found four DEPs; however, only SAA4 was significantly validated in high-RF prescreening serum. Validation of SAA4 in patients with RA also showed increased SAA4 levels in serum, with superiority over CRP as a marker of RA. Thus, SAA4 may be a new diagnostic candidate for RA, and the use of SAA4 during screening may improve prescreening diagnosis. However, further clinical studies are needed to demonstrate the applicability of SAA4 in RA diagnosis. Additionally, follow-up studies are needed to further confirm this prescreening candidate. Furthermore, MRM may also be needed to better elucidate the details of RA progression and stage markers for diagnosis.

## Figures and Tables

**Table 1:** List of differentially expressed proteins.

	Compound	*p*-value	Regulation	FC	Mass	Swiss-Prot ID
	Ig lambda-2 chain C regions	0.0000	down	−268306.0	11464.5	P0CG05
√	Cholinesterase	0.0000	down	−209972.4	68987.3	P06276
	Immunoglobulin lambda-like polypeptide 5	0.0018	down	−16107.3	23405.2	B9A064
	Hemoglobin subunit alpha	0.0029	down	−11740.1	15314.3	P69905
	Ig mu chain C region	0.0017	down	−6.5	49990.3	P01871
√	Leucine-rich alpha-2-glycoprotein	0.0078	up	2.4	38405.4	P02750
√	Serum amyloid A-4 protein	0.0017	up	3.1	14860.5	P35542
√	C-reactive protein	0.0009	up	55962.2	25209.3	P02741

The proteins labeled “√“ were subsequently analyzed using enzyme-linked immunosorbent assay (ELISA); FC, fold change.

**Table 2:** Clinical characteristics of the patients.

Subject Group		Rheumatoid Factor (Mean ± SE)	N	Sex (M/F)	Age (Mean ± SE)
Discovery set	Normal (RF < 18 IU/mL)	6.54 ± 0.88	18	4/14	65.06 ± 3.34
	High_RF (RF > 18 IU/mL)	65.69 ± 8.00	36	10/26	61.58 ± 2.41
Validation set	Normal (RF < 18 IU/mL)	5.74 ± 0.60	40	12/28	57.38 ± 1.85
	High_RF (RF > 18 IU/mL)	77.69 ± 18.20	40	12/28	57.03 ± 2.00
	RA patients	65.15 ± 9.28	40	6/34	55.43 ± 1.81

RA, rheumatoid arthritis; RF, rheumatoid factor; N, number of samples; M, male; F, female; SE, standard error.

## Supplementary Materials

The following are available online: Table S1: Peptide sequence in normal pooled serum, Table S2: Peptide sequence in high-RF pooled serum, Table S3: List of upregulated proteins in the high-RF group, Table S4: List of downregulated proteins in the high-RF group.
